# Evaluating the Use of a Generic Three-Dimensionally (3D) Printed Abdominal Aortic Aneurysm Model as an Adjunct Patient Education Tool

**DOI:** 10.7759/cureus.8533

**Published:** 2020-06-09

**Authors:** Manveer Khural, Ravindra Gullipalli, Adam Dubrowski

**Affiliations:** 1 Medicine, Memorial University of Newfoundland, St. John's, CAN; 2 Radiology, Memorial University of Newfoundland, St. John's, CAN; 3 Health Sciences, Ontario Tech University, Oshawa, CAN

**Keywords:** 3d printing, patient education, abdominal aortic aneurysm (aaa)

## Abstract

An abdominal aortic aneurysm (AAA) is a serious medical condition that requires invasive surgery or endovascular treatment with stent grafts. This procedure is primarily carried out by vascular surgeons and interventional radiologists. Current methods of educating patients about their procedure have been inadequate, causing unnecessary stress in patients who have this condition and seek treatment.

In this study, we evaluate a three-dimensionally (3D) printed AAA model to use as an adjunct patient education tool, thus allowing patients to make a more knowledgeable decision when providing informed consent. The physical attributes and realism of the model are evaluated through the use of a quantitative and qualitative survey completed by physicians at St. Clare’s Mercy Hospital in St. John’s, Newfoundland. These physicians are referred to as “Experts” in our study and also rate and comment on the necessity of having patient-specific versus generic 3D AAA models for patient education purposes.

The aim of this study is to determine whether our 3D printed AAA model is ready to be used as an adjunct patient education tool and to seek suggestions for improvements that can be made in the model. Furthermore, having generic 3D AAA models would significantly decrease healthcare costs as compared to patient-specific models. Thus, we also investigate if generic models would suffice from the perspective of the physicians.

## Introduction

In recent years, the accessibility of three-dimensional (3D) printing has allowed for advancements in patient care and simulation-based medical education (SBME) within academia and clinical practice [1]. Interventional radiology, for example, can utilize such technology to extract patient-specific vasculature from Digital Imaging and Communications in Medicine (DICOM) scans and rapidly prototype models for the rehearsal of high-acuity procedures [2,3]. Vasculature models can then be used for patient education purposes, or more commonly for medical student and resident training. They are often used by surgeons to plan and practice complex procedures, such as endovascular repair of aneurysms [2].

The purpose of this study is to focus on evaluating the use of a 3D printed abdominal aortic aneurysm (AAA) model as an adjunct patient education tool. AAA pathologies are described as a dilation of the abdominal aorta due to a combination of a weakened arterial wall and highly pressurized blood flow [4]. The enlarged section of the artery is highly susceptible to rupture without warning, which leads to massive internal bleeding and mortality rates close to 90% [5,6]. A total of 20,000 Canadians are estimated to be diagnosed with a AAA each year, and approximately 1,244 deaths linked to such conditions were reported in Canada between 2009 and 2013 [7,8]. To prevent the rupture of a AAA, a procedure called endovascular aortic repair stent grafting (EVAR) is performed. The purpose of this procedure is to create an artificial tunnel for blood flow within the aneurysm sac. This is achieved through the insertion of a series of synthetic tubes that have metal mesh supports in the aneurysm sac and securing them to the proximal and distal part of the sac [9]. This procedure is often carried out under fluoroscopic guidance by vascular surgeons and interventional radiologists along with their residents in training [3]. To improve success rates of such procedures, increased SBME is required prior to performing the procedure on an actual patient. The 3D printing of AAA models provides an efficient and risk-free way to practice EVAR, thereby reducing the risk of patient harm during the actual procedure [9]. In addition, the use of 3D printed models for SBME or patient education is a cost-effective solution to commercially available high-fidelity models that are rarely used due to their cost-prohibitive nature [9]. Furthermore, pre-operative planning and training on such cost-effective models can significantly reduce operating times, increasing the procedural success rate, as well as improve patient outcomes [2,10].

Current literature has shown the usefulness of a 3D printed AAA model for the training of interventional radiologists, vascular surgeons, and their medical residents. However, few studies have evaluated the usefulness of such models for patient education. A study by Nilsson et al. at a university hospital in Sweden showed that traditional means of patient education for an AAA is not sufficient and does not meet the patient’s learning needs, leading to unnecessary distress. Patients revealed that they were using the internet and other sources, such as anecdotal information from family and friends, to gain a better understanding of their condition and proposed treatment [11]. Thus, there is a need to improve the quality of education provided to patients with an AAA.

Previous research has shown that using 3D models as an adjunct to traditional methods can increase the patient’s understanding of their condition and make them more knowledgeable about potential complications and the expected results [12]. This puts the patient in a much better position when providing informed consent and promotes a patient-centric approach to healthcare. To date, studies evaluating the use of 3D models to facilitate the education of AAA patients have been limited. A project by Eisenmenger and colleagues assessed the utility of 3D printed patient-specific AAA models by measuring the patient’s understanding of their vascular condition before and after they had a chance to handle a personalized 3D model of their AAA. In addition to having the AAA model, the patients were also given a 3D printed model of an abdominal aorta with normal anatomy. Results showed an improvement in the patient’s understanding of their condition when the models were used as an adjunct to physicians just discussing the condition with their patients [12]. Additionally, the potential of 3D printed models for patient education can be seen in studies that focus on non-vascular pathologies, and more commonly, ones that require an understanding of anatomy that is hard to achieve through imaging and verbal discussion [13,14]. For instance, a study looked at the added benefit of using 3D printed models for educating patients on the treatment of their renal or prostate cancer. This study demonstrated that patients have a greater understanding of their anatomy, disease, and surgical treatment plan when given a 3D model to look at compared to imaging alone [13].

Given the potential of 3D printed AAA models as an adjunct patient education tool, this study aims to evaluate a generic 3D printed AAA model for its use in patient education [12-14]. In addition, the importance of a patient-specific AAA model is also assessed in this study. Both evaluations are accomplished through a survey that is completed by physicians who are referred to as “Experts” in this study.

The two objectives of this study are (a) evaluation of the efficacy and realism of a previously printed 3D AAA model and (b) assessing the importance of having a patient-specific 3D AAA model.

## Materials and methods

The 3D printed AAA model used in this study was a re-print of the AAA pathology model used in a study by Goudie et al. [3]. The model was printed in TPU95A (NinjaFlex Cheetah, Fenner Drives, Inc., Hessle, UK) on an Ultimaker 3 3D printer. The print settings were a print temperature of 235 degrees Celsius, a layer height of 0.15 mm, and a print speed of 25 mm/s. Total thermoplastic polyurethane (TPU) and polyvinyl alcohol (PVA) used were 88 g ($0.05/g) and 116 g ($0.12/g), respectively. It took approximately five hours to digitally clean up the CT angiogram scan, such as removing artefacts from the digital model, and 15 minutes of post-printing clean up to produce the first model, at an internal cost of $23.72/hour. The total cost of production of this first 3D printed AAA model when combining the material and labour cost was $142.85. Production of repeat models would not involve digital modifications and would thus cost $24.25 to make.

**Figure 1:**
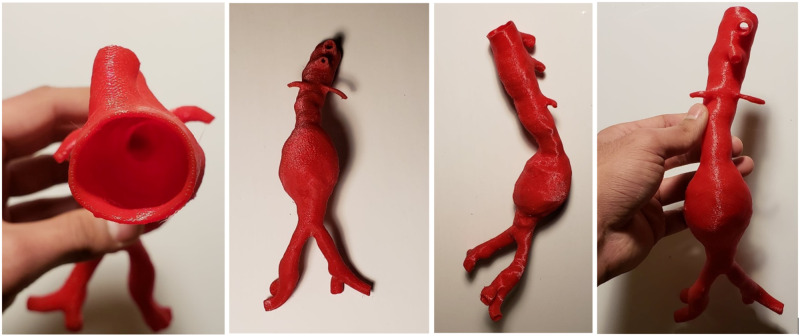
The 3D printed abdominal aortic aneurysm model as viewed from different angles

**Figure 2:**
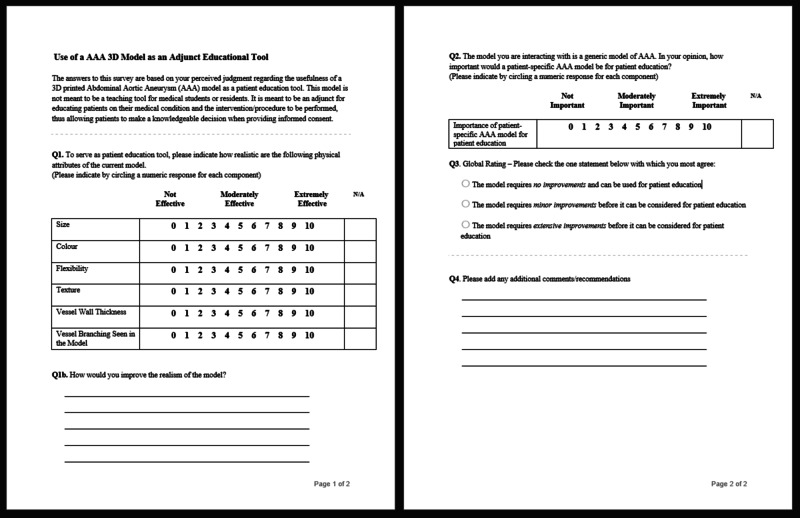
The survey completed by the Experts

The 3D printed AAA model (Figure 1) and product evaluation surveys (Figure 2) were distributed to a few Experts at St. Clare’s Mercy Hospital in St. John’s, Newfoundland. A total of 10 Experts completed the survey by handling the same 3D printed AAA model produced by the MUN MED 3D lab at Memorial University of Newfoundland. These Experts included interventional radiologists, vascular surgeons, general surgeons, and orthopaedic surgeons. Ethics board approval was not required as the surveys used to evaluate the model were completed by medical experts and not patients themselves. Therefore, the project was deemed product quality improvement.

The survey contained both quantitative and qualitative style questions, focusing on the model itself and whether it was sufficient as is, or needed improvements. The purpose of the quantitative questions was to assess the physical attributes of the model, bearing in mind its intended use for patient education. It was emphasized in the survey that this model was not meant to be a teaching tool for medical students or residents. It was meant to be an adjunct for educating patients on their medical condition and the intervention or procedure to be performed, thus allowing patients to make a knowledgeable decision when providing informed consent. All experts provided their input for the same model on its flexibility, texture, size, colour, vessel wall thickness, vessel branching seen, and the need for patient specificity. These attributes were rated on a scale of 0-10, with 0 being “not effective”, 5 being “moderately effective”, and 10 being “extremely effective”.

As mentioned previously, there are a number of studies in the current literature evaluating the benefit of patient-specific 3D printed AAA models for pre-operative training. Due to this, a question was included on the survey that asked for the necessity of having personalized or patient-specific AAA models for the purpose of patient education. This question was also rated on a scale of 0-10, with 0 being “not important”, 5 being “moderately important”, and 10 being “extremely important”. Another question on the survey asked for a global rating of the model by asking the Experts to indicate whether the model required extensive, minor, or no improvements.

Additionally, qualitative questions were used to obtain extra feedback on the model and asked for any improvements that could add to the realism of the model. It was mentioned on the survey that answers to all questions are to be based on the Expert’s perceived judgement regarding the use of our generic 3D printed AAA model as an adjunct patient education tool. After data collection was completed, the survey results were used to compile a list of modifications that could be made to the current model for its intended use in patient education. 

## Results

Quantitative data

**Figure 3:**
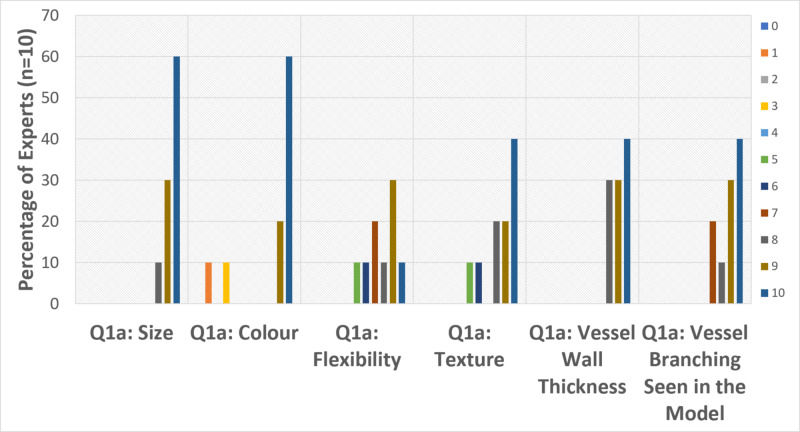
Results from Q1a: indicate how realistic are the following physical attributes of the model

The results from the survey helped us understand the improvements that can be made to the model for its use in patient education. The first question (Q1a) asked the Experts to rate the level of realism for different physical attributes of the model (Figure 3). With respect to the size of the model, there was little variability in the results as none of the respondents rated this attribute lower than 8, with more than half of the Experts rating it as a 10 or “extremely effective”. Vessel wall thickness followed a similar trend to size, with all Experts rating it above an 8, and close to half of the Experts rating it as a 10. Vessel branching seen in the model also received a positive rating of 7 and above by all Experts. Texture was not rated as favourably, receiving a rating of 5 and above by all Experts, making it closer to being “moderately effective”. Ratings for flexibility and colour showed the most variability in response. Flexibility was rated above a 5 by all Experts; however, ratings were spread out almost evenly across this range of responses. Finally, colour had somewhat of a polarized rating. It was rated as “extremely effective” by eight of the Experts; however, two Experts rated it as “not effective”.

**Figure 4:**
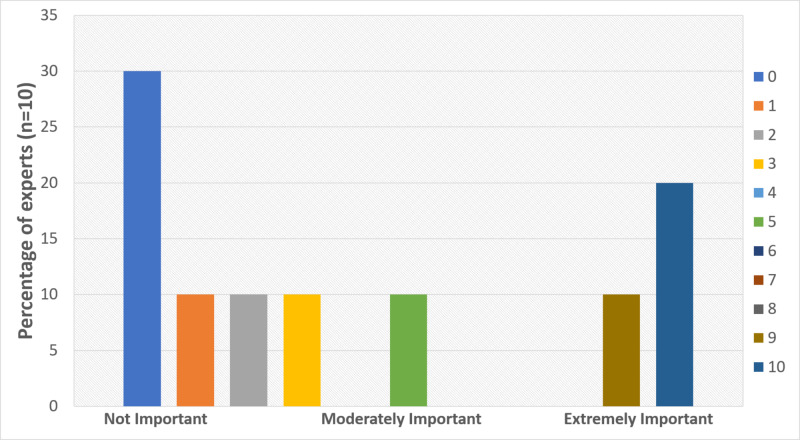
Results from Q2: importance of patient-specific abdominal aortic aneurysm model for patient education

Q2 in the survey asked the Experts to indicate the importance of patient-specific AAA models for patient education (Figure 4). There was high variability in the responses we received, with 30% of the Experts indicating a rating of 0 or “not important”, while 20% rating it a 10 or “extremely important”. However, the majority of the responses lay between “not important” and “moderately important”.

**Figure 5:**
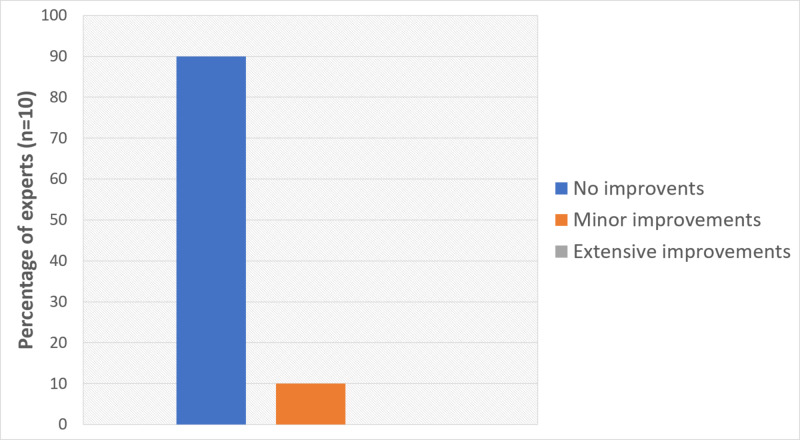
Results from Q3: global rating

Finally, the global rating (Q3) of our model was very positive, with nine out of ten Experts indicating that no improvements were required in the model and one Expert indicating minor improvements (Figure 5).

Qualitative data

Several comments were made by the Experts regarding improvements that could be made to the realism of the AAA model as asked in Q1b of the survey and Q4 that asked for any additional recommendations. Table 1 summarizes these comments and recommendations, along with the number of Experts giving similar suggestions in brackets next to each statement.

**Table 1:** Summary of results from Q1b and Q4 AAA, abdominal aortic aneurysm; EVAR, endovascular aortic repair

Expert comments and recommendations
The model is sufficient as is (4)
Increase flexibility, especially of the smaller vessels (3)
Add the iliac arteries completely into the groin level (2)
Include (if applicable) an atheromatous plaque or thrombus as part of the model (2)
Soften texture and enlarge the model a little (1)
Make the coeliac axis lie at 90 degrees to the axis of the aorta and add inferior mesenteric artery (1)
Make a model with EVAR to show patients what their AAA will look like after repair (1)
A patient-specific AAA model could be used and compared to generic non-AAA model in patient education (1)
Value of the model is likely greater for medical education than patient education. Increasing flexibility, softening texture, and enlarging the model a little could improve it for medical education and training such as suturing (1)
The model is an excellent resource for patient education (1)

## Discussion

Based on the survey results, the overall impression of the model from the Experts was positive, as indicated by their responses to the global rating. With respect to the realism of the model, there are several physical attributes that can be improved, most notably flexibility, colour, and texture. In addition, multiple recommendations include the inclusion of the iliac arteries into the groin level and possibly the presence of atheromatous plaque inside the model. Other recommendations made by the experts were the inclusion of inferior mesenteric artery and making the coeliac axis at a right angle to the axis of the aorta.

Furthermore, the Experts suggested that it would also be beneficial to have anatomically normal 3D printed models of an abdominal aorta and compare it to either a generic 3D printed AAA or a patient-specific AAA model. Additionally, it was recommended that it would be useful to have a post EVAR aorta model as well, as it would give the patient a more comprehensive picture of their treatment. A combination of these three models could have the greatest potential to improve a patient’s understanding of their condition and subsequent repair, including any complications and unforeseen results. Since the purpose of this 3D AAA model is to use it as an adjunct patient education tool, recommendations to enlarge the model for suturing purposes are not applicable. However, this would be useful if designing the model for the purpose of practicing surgical skills such as suturing for medical students and residents, as was also mentioned by the Expert that provided this comment.

This study obtained expert feedback prior to patient feedback because not only would it assist in evaluating the use of the 3D model for patient education, but would also give us valuable insight into the anatomical accuracy and appearance of the model itself. Subsequently, this would allow for necessary changes to future iterations of the model. Furthermore, it was also important to get a physician’s perspective on the importance of having patient-specific 3D AAA models as opposed to generic AAA models.

The results indicated that the generic 3D AAA model is an adequate adjunct to more traditional methods of informing patients of their condition. However, based on the survey results, the importance of having patient-specific models is not clear, although the results indicated that most experts rated patient specificity of the model as having little importance. Thus, further research needs to be conducted to explore the need for personalized AAA models in patient education. Studies evaluating the patient perspective could be a potential next step. The advantage of using generic 3D AAA models over patient-specific ones is the lower cost associated with using these models, considering the fact that close to 20,000 Canadians are diagnosed with an AAA each year [8]. It is estimated that the first print of the model used in this project costs $142.85 to make and could be used indefinitely if only done so for patient information and to obtain informed consent. Any subsequent generic models based on the parameters of the first printed model would only cost $24.25. If personalized models are not necessary, then using generic models would save healthcare costs as physicians would be using the same model as an adjunct educational tool for all their patients, as opposed to printing a new model for each patient.

Furthermore, generic AAA models can be widely distributed to healthcare centres. The benefit being that centres without nearby access to 3D printing technology, such as hospitals in a rural setting, could also make use of these models for informing patients about their condition and obtaining informed consent in a timely manner. Thus, the benefit of these models would be greatest in areas where a large proportion of the patient population lives far away from tertiary care centres. This creates a special need for the models in such areas, which include most of Atlantic Canada, especially Newfoundland and Labrador. Additionally, one way to individualize patient care without producing patient-specific models would be to produce multiple different types of generic models, each representing a common AAA pathology. Patient-specific models could also be produced on an as needed basis for more complex AAA cases, provided the health centre has access to 3D printing technology and the physician can make use of the model without too much delay in educating the patient.

This project reveals the benefit of a generic 3D printed AAA model for patient education from a physician and healthcare perspective. Future projects should address this from a patient perspective, since these models have the potential of reducing patient anxiety levels, making them feel more involved in their care, and promoting shared decision making. Furthermore, it could potentially lead to changes in procedure that are more suited to the individual patient [11,14]. Additionally, future projects could probe further into the value of having patient-specific models, as well as anatomically normal and post EVAR models. The role of virtual 3D models in AAA patient education could also be evaluated, as opposed to the real 3D models used in this study.

## Conclusions

An AAA is a life-threatening condition that requires intervention by surgery or endovascular repair (EVAR) performed by vascular surgeons and interventional radiologists. Traditional methods of patient education for this condition have been insufficient, leading to additional stress for patients. Given the potential of 3D printed AAA models, a generic 3D AAA model was evaluated to determine whether it was ready to be used as an adjunct patient education tool. Additionally, the importance of having patient-specific models of AAA compared to generic models was addressed. The results highlighted a few physical attributes of the model that could be improved in future iterations, namely flexibility, colour, and texture. The importance of patient-specific AAA models did not seem to be high, although further research may be required. The use of these models as adjunct patient education tools is promising and could be a cost-effective solution if generic AAA models are used.
